# Fragility of randomized trials supporting cancer drug approvals stratified by approval pathway and review designations

**DOI:** 10.1002/cam4.4029

**Published:** 2021-07-28

**Authors:** Brooke E. Wilson, Alexandra Desnoyers, Michelle B. Nadler, Ariadna Tibau, Eitan Amir

**Affiliations:** ^1^ Princess Margaret Cancer Centre Department of Medical Oncology University of Toronto Toronto ON Canada; ^2^ University of New South Wales Kensington NSW Australia; ^3^ Oncology Department Hospital de la Santa Creu i Sant Pau Institut d’Investigació Biomèdica Sant Pau, and Universitat Autònoma de Barcelona Barcelona Catalonia Spain

**Keywords:** accelerated approvals, FDA approvals, fragility index, trial robustness

## Abstract

**Background:**

It has been suggested that the results from fragile trials are less likely to translate into benefit in routine clinical practice.

**Methods:**

We searched the Food and Drug Administration (FDA) archives to identify drug approvals for solid organ malignancies between 2010 and 2019. We calculated the Fragility Index (FI) supporting each approval, using methods to account for time‐to‐event. We compared FI and trial and approval characteristics using Mann‐Whitney U and Kruskal‐Wallis test. Using logistic regression, we examined study characteristics associated with withdrawal of consent or lost to follow‐up (WCLFU) exceeding the calculated FI.

**Results:**

The median FI among 125 included studies was 23 (range 1–322). The FI was ≤10 in 35 studies (28%), 11–20 in 21 (17%), and >20 in 69 (55%). The median FI/Nexp was 7.7% (range 0.1–51.7%). The median FI was significantly lower among approvals processed through the accelerated vs regular pathway (5.5 vs 25, *p* = 0.001), but there was no difference in median FI/Nexp. The WCLFU exceeded FI in 42% of studies. Overall survival endpoints were more likely to have a WCLFU exceeding FI (OR 3.16, *p* = 0.003). WCLFU exceeding FI was also associated with a lesser magnitude of effect (median HR 0.69 vs 0.55, *p* < 0.001). In a sensitivity analysis including only studies with 1:1 randomization, 51% of studies had WCLFU >FI.

**Conclusion:**

The median FI among all trials was 23, and WCLFU exceeded FI in 42%. Comparative trials in solid tumors supporting approval through the accelerated pathway are more fragile compared to trials approved through the regular pathway, an observation likely explained by a lower sample size in the experimental arm.

## Graphical abstract

Trials in solid tumors supporting accelerated approval are more fragile compared to regular approvals. This finding supports the need for post‐marketing trials or real‐world analyses to ensure the benefit observed in clinical trials is robust and reproducible.

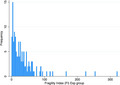

## 1 INTRODUCTION

The US Food and Drug Administration (FDA) has developed expedited review pathways and designations for approval of drugs for diseases with high unmet need.^1^ Drugs can be approved either through a regular or accelerated regulatory pathway. Regulatory approval may also be granted using an expedited review designation (fast‐tracked, breakthrough, priority review). These various pathways and designations are summarized in Table S1. The accelerated regulatory pathway allows more rapid approval of medications based on a surrogate endpoint with a reasonable likelihood of predicting clinical benefit. Most drugs processed through an accelerated regulatory approval must then fulfill post‐approval requirements including additional trials or safety analyses.^2^ Breakthrough therapies are designed to expedite the approval process for drugs that demonstrate a substantial improvement over current available therapies, while Fast Track approvals are for drugs to treat serious conditions with unmet medical need. We hypothesized that accelerated approvals, breakthrough and fast‐track designations may allow for less robust (i.e. more fragile) clinical trials to support drug registration, speeding the time to market. In contrast, priority review simply implies a commitment to rapid processing of the application (within 6 months) and is unlikely to be correlated with less robust results.

The Fragility Index (FI) is a metric quantifying the statistical robustness of randomized controlled trials (RCTs).^3^ The FI quantifies the internal reliability of clinical trials by estimating the number of events needed to change a statistically significant result to non‐significant. In contrast to a p‐value which relates to the probability that the observed results are no different between comparison groups, the FI quantifies the difference in terms of the number of events required to change a trial from positive to negative. An FI of 10 in a study with 200 participants indicates an additional 10 events in the intervention arm would render the study statistically non‐significant. Recently, journals have placed increased emphasis on reporting clinically meaningful results, encouraging a shift away from reliance on p‐values to determine the importance of results.^4^ The FI provides a clinically tangible metric of the robustness of the p‐value, in meaningful units. Furthermore, the FI can be compared directly to the number of patients withdrawing consent or being lost to follow‐up, providing further insight into the internal validity of the trial results, not otherwise captured by a p‐value.

Since its first application in 2014,^5^ the FI has been assessed in multiple areas of medicine.^6^, ^7^, ^8^, ^9^, ^10^, ^11^ However, in oncology, FI was previously calculated by dichotomizing the final event data without accounting for the time‐to‐event.^12^ In cancer, where the benefit of a drug is often measured by its ability to prolong life and/or delay disease progression or relapse, ignoring the time‐to‐event occurrence can over‐estimate the fragility of trials, as demonstrated previously.^13^, ^14^, ^15^ We have previously developed alternative methods for calculating the FI.^3^ Among a subset of tumor types, estimated median FI was 28.^3^

The objectives of this study are three‐fold: (1) to calculate the FI of all comparative trials supporting solid tumor drug registration between 2010–2019; (2) to perform a stratified analysis of FI by review pathway (accelerated vs regular) and by expedited review designation (fast‐track, break‐through or rapid review); and (3) to examine characteristics associated with studies where patient withdrawal of consent or loss to follow‐up (WCLFU) exceeds the FI.

## 2 METHODS

### 2.1 Study selection and data collection

We searched the FDA archives^16^ to identify RCTs supporting drug approvals for solid organ malignancies (excluding lymphoma) between January 2010 and December 2019. Both initial approvals and expanded indications were included, provided that the expanded indication was based on new trial results. Only studies based on trials with comparative data were included, as the FI cannot be calculated for non‐comparative data. For each identified RCT we extracted the following: tumor site, year of approval, the number of patients, randomization ratio, number of events, the hazard ratio (HR) for the outcome supporting approval, the regulatory approval pathway (regular vs accelerated) and any rapid review designations (breakthrough, fast track, priority review). The class of drug was grouped into immunotherapy, chemotherapy, monoclonal antibodies, targeted therapies (including PARP inhibitors, CDK4/6 inhibitors, mTOR inhibitors and antibody‐drug conjugates), tyrosine kinase inhibitors (TKIs), androgen receptor blockers and other. The number of patients who WCLFU was extracted from the CONSORT diagram. As WCLFU was not always reported clearly, we also extracted the number of patients who discontinued study drug for any reason other than progression, death, adverse event or completion of planned therapy (henceforth referred to as early drug discontinuation) from the CONSORT diagram.

### 2.2 Data synthesis and statistical analysis

We applied the FI framework developed by Walsh et al,^5^ modified for time‐to‐event data. We reconstructed survival tables from the published Kaplan‐Meier Curves using the Parmar Toolkit^17^ ensuring estimates of effect size and power were consistent with the primary analysis of the respective trials. Then, we calculated the number of additional events in the experimental group that would result in a non‐significant effect for the endpoint supporting drug approval. In studies with equal randomization, the FI in the experimental arm closely approximates the FI in the control arm. However, for studies with unequal randomization, we present the FI for the experimental (larger) arm. All data extractions and calculations were performed by BW, and a sample of 70 studies were verified by a second author AD to ensure reliability of data extraction (r = 0.99 between BW and AD). For studies with dichotomous outcomes, we applied the original Walsh methodology.^5^ If the approval was based on multiple significant endpoints, a hierarchy was applied with FI calculated preferentially for primary over secondary endpoints, and overall survival (OS) over other co‐primary endpoints. If multiple trials (or subgroups) were used for a given drug approval, the trial (or subgroup) with the highest FI (i.e. most robust) was chosen. We then calculated the FI as a proportion of the experimental group size (FI/Nexp) to provide a standardized measure between studies accounting for sample size.

We compared the association between FI and FI/Nexp with trial characteristics and the approval or rapid review pathway using Mann Whitney *U* (2 groups) and Kruskal‐Wallis test (>2 groups). Trends over time were assessed through log transformation of the FI or FI/Nexp (to normalize the data), followed by linear regression. The association between trial characteristics and trials where WCLFU exceeds FI was examined using univariable logistic regression. HR were log transformed for statistical testing to ensure linearity of effect size. Multivariable analyses were not planned as the small number of comparative trials supporting accelerated approval did not allow for adequate fitting of a multivariable model. We then performed two sensitivity analyses. First, we examined trial characteristics associated with early drug discontinuation being greater than the FI. Second, we included only studies with 1:1 randomization and re‐examined trial characteristics associated with WCLFU exceeding FI. All analyses were performed using STATA version 12.0 (StataCorps LP). Statistical significance was defined as *p* < 0.05. No corrections were applied for multiple significance testing.

## 3 RESULTS

**FIGURE 1:**
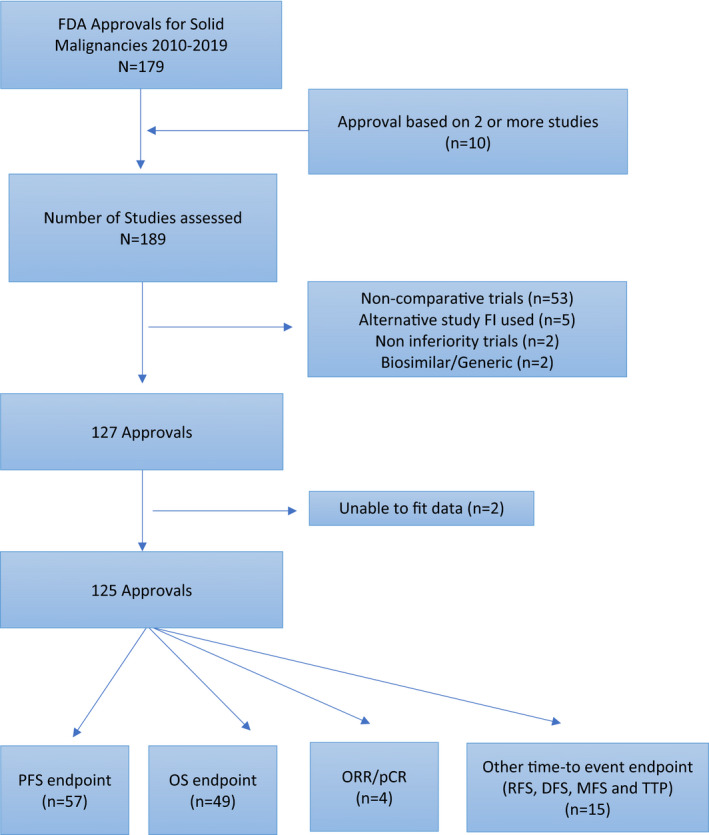
Schema for study inclusion. KM‐Kaplan‐Meier; PFS‐progression free survival; OS‐overall survival; RFS‐relapse free survival; DFS‐disease free survival; MFS‐metastasis free survival; TTP‐time to progression; ORR‐objective response rate; pCR‐pathological complete response

We identified 179 drug approvals (42 accelerated and 137 regular). After excluding non‐inferiority, biosimilar and non‐comparative studies, 127 approvals and their associated trials were selected. Among those processed through the accelerated pathway, 34 (81%) were excluded from further analysis as they were based on single arm or non‐comparative studies (Table S1). In contrast, among the 137 studies processed through the regular approval pathway, only 20 (14.5%) were excluded (18 based on study design being either non‐inferiority, non‐comparative or biosimilar, 1 because the data could not be fitted adequately to the Kaplan‐Meier curve, and 1 because the survival curve was not available). Therefore, 125 studies were included in the remaining analysis (Figure 1). Among the included studies in our cohort, there were no instances identified where a single trial resulted in multiple approval indications.

**FIGURE 2:**
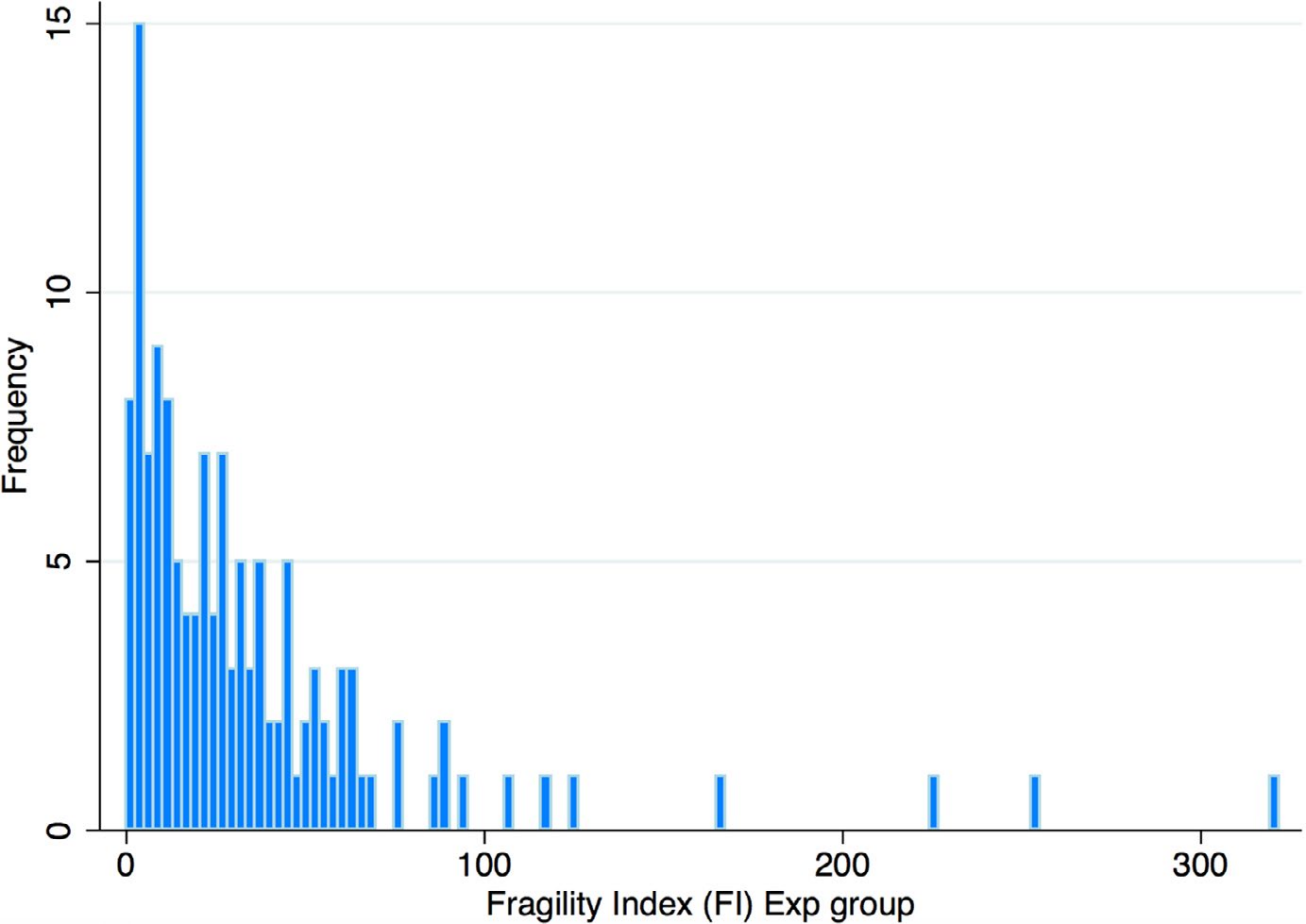
Frequency of fragility index of trials in solid malignancy gaining food and drug administration approval between 2010–2019 (n = 125)

The median FI among all 125 included studies was 23 (range 1–322) (Figure 2). Characteristics of the included studies are presented in Table S2. The FI was ≤10 in 35 studies (28%), 11–20 in 21 studies (17%), and >20 in 69 studies (55%). Of the 125 comparative studies included, 117 were processed through regular approval (116 with time‐to‐event endpoints, 1 with ORR) while only 8 (6.4%) were processed through accelerated regulatory approval (5 with time‐to‐event endpoints, 2 with ORR as the primary outcome and 1 with pathological complete response [pCR]). The FI and the FI/Nexp for different trial and approval characteristics is reported in Table 1. The median FI among trials processed through accelerated approval (n = 8) was significantly lower than the included studies processed through regular approval (n = 117) (5.5 vs 25, *p* = 0.001). The median FI/Nexp among all included studies was 7.7% (range 0.1 to 51.7%), and FI/Nexp was similar between regular and accelerated approvals (7.8% vs 7.3%, *p* = 0.60) (Table 1).

**TABLE 1:** Median fragility index by trial characteristics

Trial and Approval Characteristics (n = 125)	Fragility Index Median (range)	*p* value	FI/Nexp (%) Median ± SD	*p* value
Regulatory Approval Pathway		0.0012		0.6
Regular (n = 117)	25 (1–322)		7.8 (0.1–51.7)	
Accelerated (n = 8)	5.5 (2–22)		7.3 (1.8–16.7)	
Breakthrough Therapy Designation		0.17		0.27
No (n = 77)	22 (1–322)		7.1 (0.1–51.7)	
Yes (n = 30)	30.5 (5–108)		7.8 (2.5–38.7)	
N/A^b^ (n = 18)	21 (1–68)		8.2 (0.4–30.4)	
Fast Track Designation		0.66		0.52
No (n = 92)	23 (1–322)		7.1 (0.1–51.7)	
Yes (n = 25)	34 (2–254)		10.4 (0.6–34.2)	
N/A^d^ (n = 8)	22 (1–68)		9 (0.5–30.4)	
Priority Review Designation		0.92		0.39
No (n = 31)	26 (1–322)		7.1 (0.3–34.5)	
Yes (n = 94)	22 (1–254)		7.75 (0.1–51.7)	
Composite Rapid Review Designation^a^		0.81		0.29
No Rapid Review Designation (n = 29)	25 (1–322)		6.5 (0.3–34.5)	
Any Rapid Review Designation (n = 96)	22 (1–256)		7.9 (0.1–51.7)	
Endpoint		<0.001		<0.001
Other^c^ (n = 76)	32 (2–322)		11.7 (0.1–51.7)	
OS (n = 49)	12 (1–88)		3.9 (0.3–14.6)	
Year of Approval		0.04		0.096
2010/12 (n = 26)	22 (1–90)		8.85 (0.4–34.2)	
2013/15 (n = 34)	12 (1–117)		6.95 (0.3–44.8)	
2016/17 (n = 25)	24 (2–108)		7.6 (0.1–38.7)	
2018/19 (n = 40)	32 (3–322)		9.9 (1.5–51.7)	
Drug Class		<0.001		<0.001
Immunotherapy (n = 28)	20.5 (3–77)		6.6 (1.8–21.4)	
Chemotherapy (n = 9)	12 (2–54)		3.9 (0.4–15)	
Monoclonal Antibodies (n = 17)	6 (1–32)		2.1 (0.1–11.2)	
Androgen Receptor Blockers (n = 9)	90 (20–322)		16.5 (3.8–34.5)	
TKI (n = 34)	24.5 (2–117)		11.9 (0.4–44.8)	
Targeted (n = 25)	34 (5–87)		10.1 (1–33.3)	
Other (n = 3)	43 (18–60)		7 (2.9–51.7)	
Malignancy Site		0.0013		0.12
Breast (n = 21)	24 (2–63)		8 (0.1–18.2)	
Lung (n = 26)	27 (3–108)		11.75 (0.6–38.7)	
Melanoma (n = 15)	25 (2–93)		7.5 (1.9–21.9)	
Prostate (n = 11)	88 (12–322)		11 (3.2–34.5)	
Other (n = 52)	15.5 (1–117)		6.25 (0.3–51.7)	
Setting of Approval		0.8		0.29
Metastatic (n = 113)	23 (1–322)		7.8 (0.3–51.7)	
Neoadjuvant/Adjuvant (n = 12)	24 (2–77)		6.7 (0.1–21.2)	
Control Group		0.04		0.07
Active Control (n = 89)	23 (1–322)		7.2 (0.1–51.7)	
Placebo or BSC alone (n = 36)	33.5 (3–117)		9.1 (0.4–44.8)	

Abbreviations: BSC, best supportive care; N/A, not available; OS, overall survival; TKI, Tyrosine kinase inhibitors.

^a^ Composite Rapid Review Designation includes Breakthrough, Fast Track and/or Priority Review.

^b^ Breakthrough began in 2016, studies published prior to 2016 were not eligible for this designation.

^c^ Other includes PFS, DFS, EFS and dichotomous endpoints.

^d^ Fast‐track listings are publicly available through the FDA website for all years from 1998 until 2020, except 2011 where the data are not available (n = 8, listed as N/A).

There was no difference in the proportion of studies excluded when stratified by priority review designation (Table S3). However, a higher proportion of studies given breakthrough designation were excluded from our analysis (49.1% vs 21.4%), as a higher proportion were based on non‐comparative or single arm trials (Table S3). In contrast, the proportion of excluded studies given fast‐track designation was smaller (13.8% vs 34.7% not given fast‐track designation). Among the included studies, there was no significant difference in FI or FI/Nexp for drugs processed with any of the expedited review designations (Table 1).

The median FI was significantly lower in studies where OS was the endpoint on which approval was based (12 vs 32, *p* < 0.001), as was the FI/Nexp (11.7% vs 3.9%, *p* < 0.001). Higher FI was seen in studies where the control arm was placebo or best supportive care compared to studies with an active control (33.5 vs 23, *p* = 0.04); however, when examining the association between FI/Nexp and the type of control arm this did not meet statistical significance (7.2% vs 9.1%, *p* = 0.7). The median FI was similar between immunotherapy (20.5), chemotherapy (19), TKIs (24.5), and targeted agents (34) but lower in monoclonal antibodies (5) and significantly higher in studies of androgen receptor blockers (90) (*p* < 0.001). In keeping with these results, FI/Nexp was highest in studies of androgen receptor blockers (16.5%), but lowest in chemotherapy studies (3.9%). The median FI was higher in prostate cancer trials (88) compared to other tumor groups (*p* < 0.001). This was driven by the larger sample size of prostate cancer studies (median sample size 1195 prostate vs 519.5 for all other cancer types, *p* < 0.001). After adjusting for sample size in the experimental arm, the FI/Nexp was highest in lung (11.75%) and prostate (11%) trials.

WCLFU was reported in 117 studies. In the remaining eight studies, there was either insufficient information presented in the CONSORT diagram, or WCLFU was not clearly presented for the subgroup of interest. The median percentage of patients WCLFU among all studies was 2.9% (mean 4.2%). The association between WCLFU and FI is shown in Supplemental Figure S1. The WCLFU was higher than the calculated FI in 49 studies (42%). Study characteristics associated with WCLFU ≥ FI are shown in Supplemental Table S4 and Table 2. As a percentage of the total sample size, the WCLFU was 5.6% in the group of studies where WCLFU ≥ FI compared to 1.2% in studies where WCLFU < FI (*p* < 0.001). WCLFU ≥ FI was also associated with a smaller effect size (HR 0.69 vs 0.55, *p* < 0.001). The median sample size was quantitatively but not statistically higher in studies where WCLFU ≥ FI (658 vs 557.5, *p* = 0.20). OS endpoints were more likely to have WCLFU ≥FI (OR 3.16, *p* < 0.001). There was no association between accelerated approvals or any of the rapid review pathways and WCLFU ≥FI.

**TABLE 2:** Association between studies where WCLFU exceeds the fragility index^a^ and trial characteristics (n = 117)

	OR	95% CI	*p*
Fragility Index (median, range)	0.95	0.92–0.97	<0.001
Reported HR (median, range) (n = 114)^c^	48.13	7.13–323.50	<0.001
Number WCLFU (median, range)	1.04	1.02–1.07	<0.001
WCLFU as percent of total sample size (median, range)	1.81	1.44–2.28	<0.001
Fragility Index as proportion of experimental group size (median, range)	0.83	0.76–0.90	<0.001
Sample Size (median, range)	1	0.99–1.00	0.2
Year of Approval (n, %)			0.54^b^
2010/12 (n = 23)	1		
2013/15 (n = 31)	1.28	0.42–3.83	
2016/17 (n = 25)	1.98	0.63–6.26	
2018/19 (n = 38)	0.72	0.24–2.12	
Number of Events (median, range) (n = 107)	1	0.99–1.00	0.45
Drug Class (n, %)			
Immunotherapy (n = 26)	1		
Chemotherapy (n = 9)	0.5	0.10–2.43	0.39
Monocolonal Antibodies (n = 17)	1.8	0.52–6.44	0.34
Androgen Receptor Blockers (n = 6)	0.2	0.02–1.95	0.17
TKI (n = 31)	0.7	0.25–2.06	0.54
Targeted (n = 25)	0.3	0.09–1.04	0.06
Other (n = 3)	2	0.16–24.9	0.6
Setting of Approval (n, %)			
Metastatic (n = 106)	1		
Adjuvant (n = 11)	1.76	0.50–6.13	0.38
Malignancy Site (n, %)			
Breast (n = 21)	1		
Lung (n = 25)	0.73	0.22–2.37	0.6
Melanoma (n = 13)	1.28	0.32–5.13	0.72
Prostate (n = 8)	0.37	0.06–2.25	0.28
Other (n = 50)	0.73	0.26–2.05	0.55
Endpoint (n, %)			
Other (n = 73)	1		
OS (n = 49)	3.16	1.46–6.85	0.003
Difference in time to event outcome between intervention and control in months (median, range) (n = 94)	0.87	0.77–0.99	0.04
Regulatory Approval Pathway (n, %)			
Regular (n = 111)	1		
Accelerated (n = 6)	2.9	0.52–16.7	0.22
Breakthrough Therapy Designation (n, %)			
No (n = 71)	1		
Yes (n = 30)	0.79	0.33–1.91	0.6
N/A (n = 16)	1.37	0.46–4.05	0.57
Fast Track (n, %)			
No (n = 86)	1		
Yes (n = 24)	0.79	0.31–2.01	0.63
N/A (n = 7)	0.99	0.21–4.71	0.99
Priority Review (n, %)			
No (n = 30)	1		
Yes (n = 87)	1.34	0.57–3.15	0.5
Control Group (n, %)			
Active Control (n = 83)	1		
Placebo or BSC alone (n = 34)	1.13	0.51–2.54	0.75

Abbreviations: BSC‐ best supportive care; FU‐follow up; N/A‐not available; OR‐odds ratio; CI‐confidence interval; FI‐fragility index; OS‐overall survival; TKI‐Tyrosine kinase inhibitors.

^a^ In 49 studies, the WCLFU>FI, while in 68 studies the WCLFU<FI. See Supplemental Table S3.

^b^ *p* test for trend.

^c^ 3 excluded studies had dichotomous endpoints.

In sensitivity analysis including only studies with 1:1 randomization (n = 71), 51% of studies (n = 36) had WCLFU ≥ FI (Table S5). WCLFU ≥FI remained strongly associated with lower magnitude of effect (median HR 0.70 vs 0.57, *p* = 0.004). There was no longer any association with type of study endpoint (Table S5), and there was no association with approval type or rapid review pathways. In 76 studies (65%), the number of patients with early drug discontinuation ≥FI. Early drug termination ≥FI was also associated with OS as a primary endpoint (OR 8.43, *p* < 0.001) (Table S6).

## 4 DISCUSSION

This study includes a large dataset examining FI in oncology. The median FI among the 125 included studies was 23 (range 1–322), meaning 23 additional events would result in a non‐significant effect for the trial endpoint supporting drug approval. This is slightly lower to prior data reported in oncology examining approvals for only a subset of tumors,^3^ but higher than the median FI of 8 calculated by Walsh et al for the primary endpoint of high impact general medicine studies with dichotomous outcomes,^5^ suggesting that outcomes for solid organ malignancies may be more robust. This may be a reflection of the higher statistical power that results from the use of time‐to‐event rather than dichotomous outcomes which form the majority of endpoints in general medicine.^18^ Moreover, in contrast to the Walsh study, all trials included in this analysis resulted in FDA approval of often costly drugs.^19^

Of note, almost 30% of drug approvals between 2010 and 2019 were supported by trials with a FI of 10 or less, this may impact the sensitivity analyses of health technology assessments, potentially rendering some drugs not cost‐effective. After adjusting for sample size in the experimental arm, the median proportion of patients in the experimental group that would need to have an alternative outcome to render the results non‐significant was less than 10% and in once case was as low as 0.1%. That one drug was granted FDA approval based on a result that would have been insignificant if the outcome had been different in 0.1% of the experimental population is concerning.

Comparative studies processed through the accelerated regulatory pathway had a lower fragility index (i.e. more fragile) than comparative studies processed through the regular approval pathway, although there was no significant difference in the fragility after adjusting for the size of the experimental arm (FI/Nexp). During our study window, only 8 of 42 (19%) drugs processed through the accelerated regulatory pathway for solid malignancies had comparative data from which FI could be calculated, as compared to 85% of those processed through the regular approval pathway. Therefore, our finding of lower FI (i.e. higher fragility) among studies processed through the accelerated approval pathway only applies to comparative studies, and cannot be generalized to non‐comparative trials supporting accelerated approval. Instead, our results highlight an important limitation of single‐arm data; internal robustness of these trials cannot be quantified easily. The differences in the proportion of approvals supported by comparative data between accelerated and regular approval pathways is itself important, and is in keeping with prior research demonstrating that drugs processed through accelerated pathways are more likely to be single‐arm and utilize ORR as the primary outcome.^2^ Together, these findings support the requirement for post‐approval trials in drugs processed through the accelerated pathway even if based on comparative data, to confirm benefit and demonstrate robust results.

We also found a higher proportion of studies given break‐through designation were based on non‐comparative data and excluded from our analysis. In contrast, the proportion of excluded studies was similar for priority review designation, and lower for those given fast‐track designation. FI was similar regardless of whether any expedited review designations were used, providing some reassurance that drugs used to treat solid malignancies given expedited review designations (priority review, fast track and breakthrough) are as robust as those processed without expedited designations, when initially supported by comparative data. However, these results cannot be generalized to non‐comparative studies.

Another concern is that the WCLFU was greater than the FI in 42% of studies, and early drug discontinuation exceeded FI in 65% of studies. This is similar to the results by Walsh et al., evaluating studies in general medicine.^5^ Trials where WCLFU exceeds the FI should be interpreted with caution, as uncaptured events in censored patients could render the results non‐significant. Prior research in general medicine has shown that the median percentage of participants lost to follow‐up is 6%, but that the quality of reporting is inconsistent.^20^ We found that median percentage of patients WCLFU among all studies was 2.9% (mean 4.2%), supporting the high quality of oncology trials leading to FDA approvals. However, patients rarely withdraw or are lost to follow‐up from clinical studies if they are doing well, and in advanced cancer, rates of progression among censored patients are higher than in those who remain on study.^21^ In a simulation study in general medicine, varying the assumptions regarding the event rate in patients lost to follow‐up caused 17% to 58% of positive studies to become non‐significant.^20^ While simulations were beyond the scope of this paper, our finding that 42% of studies had WCLFU >FI suggests a similar proportion of positive studies in oncology would become non‐significant if event rates are higher among those WCLFU, especially if there were differences in the proportion of patients censored in the experimental and control groups.^22^ As such, trials where WCLFU exceeds the FI may over‐estimate the benefits of treatment, may be less likely show benefit in routine clinical practice, and may have inferior cost‐effectiveness.

In this study, FI is calculated based on the number of additional events in the experimental arm required to make the results insignificant. In trials with unequal randomization, the FI will differ as a function of experimental or control group size. Therefore, comparing FI to WCLFU in the total sample may overestimate the number where WCLFU > FI in studies with unequal randomization. However, in sensitivity analysis excluding studies with unequal randomization, we found 51% of studies with WCLFU exceeding FI, suggesting that prior estimates may be conservative.

The performance of drugs in clinical trials generally exceeds results seen in real‐world practice. Studies have demonstrated an efficacy‐effectiveness gap for hepatocellular carincoma,^23^ lung cancer,^24^, ^25^ prostate cancer,^26^ breast and hematological malignancies.^27^ The efficacy‐effectiveness gap is often attributed to the differences that exist between patients in trials and in routine practice including clinically relevant differences in age, performance status, co‐morbidities and prior and subsequent lines of therapy. These differences can result in variability in toxicity and drug tolerability as well as long‐term outcomes. Studies with low FI, where small changes in the number of events renders the results insignificant, may be more vulnerable to these differences between clinical trials and real‐world practice and may result in a higher efficacy‐effectiveness gap.

Over time, an increasing number of drugs are being processed through the expedited development or review programs.^28^ At present, only those processed through accelerated approval have mandated post‐marketing requirements. And yet, there is evidence that over 50% of approvals have not completed all post‐marketing requirements 3 years after obtaining approval,^29^ and in up to 25% the results may not be disseminated publicly.^30^ Even among accelerated approvals that have undergone confirmatory trials, only 20% demonstrated improvements in overall survival.^31^ As an example, Olaratumab was granted accelerated approval in 2016 based on phase Ib/II results demonstrating prolonged OS.^32^ Based on our calculations, the FI of this study was 5. The FDA mandated confirmatory phase III trial ANNOUNCE^33^ failed to demonstrate any improvements in OS and the approval was subsequently withdrawn. As the outcomes of trials are difficult to predict, studies with low fragility index (i.e. more fragile results) may not be preventable in the trial design stages. However, studies with low FI, regardless of whether they are processed through regular or accelerated pathways or whether they are given expedited review designations, should be prioritized for confirmatory trials, especially if the approval was based on early phase data. We would encourage the FDA to look at other metrics of trial robustness and internal validity, such as the FI, that might indicate whether a study result requires confirmation, regardless of approval pathway or expedited designation. Whether a low FI could be used to predict which studies based on early phase data are unlikely to confirm benefit in larger phase III studies remains to be seen, and ongoing research is needed.

This study has important limitations. There were a small number of comparative trials supporting accelerated approvals included in our analysis, and further research over time to expand this dataset and confirm these results is needed. Accelerated approvals based on non‐comparative data were excluded from this analysis as the FI cannot be calculated, introducing sampling bias. Therefore, the conclusions regarding lower trial robustness may only be applied to accelerated approvals based on comparative data. Studies with smaller sample size granted accelerated approval are likely to have lower FI than larger studies, if based on the same endpoint. By providing FI as a percentage of the total the experimental sample size (FI/Nexp) we have provided standardization between studies. Our results have also demonstrated that OS endpoints are more fragile than surrogate endpoints, which are more commonly used in studies processed through the accelerated regulatory pathway. Therefore, accelerated approvals still have the potential for robust results when based on surrogate endpoints. Due to the small sample size of this study, we were unable to fit a multivariable analysis. In this study, we applied the Walsh methodology adapted for time‐to‐event data using the Palmar toolkit. At least two other methodologies have been applied in oncology to examine time‐to‐event data.^14^, ^15^ While each applies similar principles, the resulting FI calculations may differ and research into comparative methodologies would be helpful. Finally, studies did not always present WCLFU data clearly, and therefore we may have underestimated the true number of patients WCLFU. Furthermore, patients may be censored from the analysis for reasons other than WCLFU, and this could also impact on the validity and robustness of results. Unfortunately, censoring rates and reasons are often not clearly reported, and improved transparency should be encouraged.^22^

## 5 CONCLUSION

The median FI among all comparative trials supporting regulatory approval in oncology between 2010 and 2019 was 23. Trials in solid tumors processed through the accelerated review pathway are more fragile compared to those processed through the regular approval pathway, an observation likely explained by a lower sample size in the experimental arm. There was no difference in the FI for studies processed through any of the rapid review pathways. In just under half of studies, the number of patients WCLFU exceeded FI, supporting the need for post‐marketing trials or real‐world analyses to ensure the benefit observed in clinical trials is robust and reproducible, regardless of approval pathway or expedited designations.

## CONFLICT OF INTERESTS

Dr. Eitan Amir reports personal fees from Genentech/Roche, personal fees from Apobiologix, personal fees from Myriad Genetics, personal fees from Agendia, outside the submitted work. No Conflict of Interest for Drs Brooke E. Wilson, Alexandra Desnoyers, Michelle B. Nadler and A. Tibau.

## ETHICS STATEMENT

As all data were publicly available, no ethics approval was sought.

## Supporting information

Supplementary Material

## Data Availability

The data that support the findings of this study are available from the corresponding author upon reasonable request.
