# Supplementary material for: Fragility of randomized trials supporting cancer drug approvals stratified by approval pathway and review designations
Source: Cancer Med. 2021 Jul 28;10(16):5405–14. doi: 10.1002/cam4.4029 (PMC8366090; doi:10.1002/cam4.4029)
Supplement: Supplementary file 1 — Supplementary Material [file CAM4-10-5405-s001.docx]

**Supplemental Table 1: Summary of the FDA review pathways and designations, and our hypothesised impact on fragility index**

| Approval or Review pathway | Objective of pathway/designation | Hypothesised impact on trial fragility index | Post-marketing requirements |
| --- | --- | --- | --- |
| Accelerated Approval | Allows for the approval of a drug that demonstrates an effect on a surrogate endpoint that is reasonably likely to predict clinical benefit, or on a clinical endpoint that occurs earlier but may not be as robust as the standard endpoint used for approval. This pathway is especially useful when the drug is meant to treat a disease whose course is long, and an extended period of time is needed to measure its effect. | Accelerated approvals may allow smaller less robust trials based on surrogate endpoints to gain approval. Therefore, we hypotheses a lower fragility index among studies processed through the accelerate approval pathway. | Yes, confirmatory post-marketing trials mandated. |
| Breakthrough designation | Expedites the development and review of drugs that are intended to treat a serious condition, and preliminary clinical evidence indicates that the drug may demonstrate substantial approval over available therapy. | As approval may be based on preliminary evidence of benefit, we anticipate lower fragility of studies given break-through designation | No post-approval requirements |
| Fast-track | Designed to facilitate the development and advance the review of drugs to treat serious conditions, and fill an unmet medical need based on promising animal or human data. Fast-tracking can get important new drugs to the patient earlier. | As approval may be based on early data from animal and humans, we would anticipate lower fragility index in studies processed through fast-track program. | No post-approval requirements |
| Rapid Review | Means the FDA aims to take action on an application within 6 months, compared to 10 months under standard review | This designation only speeds time to review. We would not anticipate any impact on the fragility index. | No post-approval requirements |

These definitions are drawn from the FDA website, available at https://www.fda.gov/drugs/development-approval-process-drugs

**Supplemental Table 2: Characteristics of Included Studies (n=125)**

|  | N =125  (n, %) |
| --- | --- |
| Regulatory Approval Pathway  Regular  Accelerated | 117 (93.6%)  8 (6.4%) |
| Breakthrough Therapy Designation  No  Yes  N/A^*****^ | 77 (61.6%)  30 (24%)  18 (14.4%) |
| Fast Track Designation  No  Yes  N/A^****^ | 92 (73.6%)  25 (20%)  8 (6.4%) |
| Priority Review Designation  No  Yes | 31 (24.8%)  94 (75.2%) |
| Composite Rapid Review Designation^***^  No Rapid Review Designation  Any Rapid Review Designation | 29 (23.2%)  96 (76.8%) |
| Endpoint  Other^******^  OS | 76 (60.8%)  49 (39.2%) |
| Year of Approval  2010/12  2013/15  2016/17  2018/19 | 26 (20.8%)  34 (27.2%)  25 (20%)  40 (32%) |
| Drug Class  Immunotherapy  Chemotherapy  Monoclonal Antibodies  Androgen Receptor Blockers  TKI  Targeted  Other | 28 (22.4%)  9 (7.2%)  17 (13.6%)  9 (7.2%)  34 (27.2%)  25 (20%)  3 (2.4%) |
| Malignancy Site  Breast  Lung  Melanoma  Prostate  Other | 21 (16.8%)  26 (20.8%)  15 (12%)  11 (8.8%)  52 (11.6%) |
| Setting of Approval  Metastatic  Neoadjuvant/Adjuvant | 113 (90.4%)  12 (9.6%) |
| Control Group  Active Control  Placebo or BSC alone | 89 (71.2%)  28.8%) |
| Fragility Index (median, range) | 23 (1-322) |
| Fragility Index as proportion of experimental group size (median, range) | 7.7% (0.1-51.7%) |
| Reported HR  (median, range) (n=121)^*^ | 0.62 (0.21-0.86) |
| Number WCLFU (median, range) (n=117)^**^ | 13 (0-600) |
| WCLFU as percent of total sample size (median, range) (n=117)^**^ | 2.88% (0-65.1%)  Mean 4.17% |
| Sample Size (median, range) | 559 (109-4804)  Mean 653.5 |

^*^ 4 studies based on dichotomous endpoints (ORR or pCR) do not present HR data

^**^ WCLFU data available for 117 of the included studies

^***^Composite Rapid Review Designation includes Breakthrough, Fast Track and/or Priority Review

^****^ Breakthrough began in 2016, studies published prior to 2016 were not eligible for this designation.

^*****^ Fast-track listings are publicly available through the FDA website for all years from 1998 until 2020, except 2011 where the data is not available (n=8, listed as N/A)

^******^ Other includes PFS, DFS, EFS and dichotomous endpoints.

**Supplemental Table 3: Potential sampling bias based on the proportion of included and excluded studies by approval pathway and review designation**

| Approval/Review Pathway | Total number of studies identified (n=179) | Number of studies included (% of total)  (n=125) | Number of studies excluded (% of total)  (n=54) |
| --- | --- | --- | --- |
| Approval Pathway  Accelerated  Regular | 42  137 | 8 (19%)  117 (85.5%) | 34 (81%)  20 (14.5%) |
| Fast-Track Designation  Yes  No  NA | 29  141  9 | 25 (86.2%)  92 (65.2%)  8 (88.9%) | 4 (13.8%)  49 (34.7%)  1 (11.1%) |
| Breakthrough Designation  Yes  No  NA | 59  98  22 | 30 (50.8%)  77 (78.6%)  18 (81.8%) | 29 (49.1%)  21 (21.4%)  4 (18.2%) |
| Priority Review Designation  Yes  No | 139  40 | 94 (67.6%)  31 (77.5%) | 45 (32.4%)  9 (22.5%) |

**Supplemental Table 4: Association Between Studies Where WCLFU Exceeds the Fragility Index and Trial Characteristics (n=117)**

|  | Loss to follow up greater than FI  (n=49) | Loss to follow up less than FI (n=68) |
| --- | --- | --- |
| Fragility Index (median, range) | 10 (1-93) | 34.5 (3-322) |
| Reported HR (median, range) (n=114)^**^ | 0.69 (0.46-0.86) | 0.55 (0.21-0.84) |
| Number WCLFU (median, range) | 31 (7-600) | 5 (0-222) |
| WCLFU as percent of total sample size (median, range) | 5.6% (0.3-65.1%) | 1.2% (0-14.7%) |
| Fragility Index as proportion of experimental group size (median, range) | 3.99% (0.08-27.6%) | 12.1% (1.1-51.7%) |
| Sample Size (median, range) | 658 (123-4804) | 557.5 (117-1509) |
| Year of Approval (n, %)  2010/12 (n=23)  2013/15 (n=31)  2016/17 (n=25)  2018/19 (n=38) | 9 (18.3%)  14 (28.6%)  14 (28.6%)  12 (24.5%) | 14 (20.6%)  17 (25.0%)  11 (16.2%)  26 (38.2%) |
| Number of Events (median, range) (n=107) | 320 (56-884) | 279.5 (81-1077) |
| Drug Class (n, %)  Immunotherapy (n=26)  Chemotherapy (n=9)  Monocolonal Antibodies (n=17)  Androgen Receptor Blockers (n=6)  TKI (n=31)  Targeted (n=25)  Other (n=3) | 13 (26.5%)  3 (6.1%)  11 (22.4%)  1 (2.0%)  13 (26.5%)  6 (12.2%)  2 (4.1%) | 13 (19.1%)  6 (8.8%)  6 (8.8%)  5 (7.3%)  18 (26.5%)  19 (27.9%)  1 (1.5%) |
| Setting of Approval (n, %)  Metastatic (n=106)  Adjuvant (n=11) | 43 (87.8%)  6 (12.2%) | 63 (92.6%)  5 (7.4%) |
| Malignancy Site (n, %)  Breast (n=21)  Lung (n=25)  Melanoma (n=13)  Prostate (n=8)  Other (n=50) | 10 (20.4%)  10 (20.4%)  7 (14.3%)  2 (4.1%)  20 (40.8%) | 11 (16.2%)  15 (22.1%)  6 (8.8%)  6 (8.8%)  30 (44.1%) |
| Endpoint (n, %)  Other (n=73)  OS (n=49) | 22 (44.9%)  27 (55.1%) | 49 (72.1%)  19 (27.9%) |
| Difference in time to event outcome between intervention and control in months (median, range) (n=94) | 2.95 (1.2-15.3) | 4.75 (1.3-24.3) |
| Approval Pathway (n, %)  Regular approval (n=111)  Accelerated Approval (n=6) | 45 (91.8%)  4 (8.2%) | 66 (97.1%)  2 (2.9%) |
| Breakthrough Therapy Designation (n, %)  No (n=71)  Yes (n= 30)  N/A (n=16) | 30 (61.2%)  11 (22.4%)  8 (16.3%) | 41 (60.3%)  19 (27.9%)  8 (11.8%) |
| Fast Track (n, %)  No (n=86)  Yes (n=24)  N/A (n=7) | 37 (75.5%)  9 (18.4%)  3 (6.1%) | 49 (72.1%)  15 (22.1%)  4 (5.9%) |
| Priority Review (n, %)  No (n=30)  Yes (n=87) | 11 (22.4%)  38 (77.6%) | 19 (27.9%)  49 (72.1%) |
| Control Group (n, %)  Active Control (n=83)  Placebo or BSC alone (n=34) | 34 (69.4%)  15 (30.6%) | 49 (72.1%)  19 (27.9%) |

Abbreviation: BSC, best supportive care; FU, follow up; N/A, not available; OR, odds ratio; CI, confidence interval; FI, fragility index; OS; overall survival; TKI, Tyrosine kinase inhibitors.

^*^ p test for trend

^**^ Only 114 included studies as 3 had dichotomous endpoints. While the HR are reported based on the trial results, the OR and P values are reported for the log transformed HR.

**Supplemental Table 5:** **Association Between Studies where WCLFU Exceeds the Fragility Index and Trial Characteristics among studies with only 1:1 randomization (n=71)**

|  | WCLFU > FI  (n=36) | WCLFU <FI (n=35) | OR | 95% CI | P |
| --- | --- | --- | --- | --- | --- |
| Fragility Index (median, range) | 9.5 (1-93) | 26 (3-125) | 0.96 | 093-0.99 | **0.01** |
| Reported HR (median, range) (n=70)^**^ | 0.70 (0.46-0.86) | 0.57 (0.21-0.84) | 26.7 | 2.77-258.0 | **0.004** |
| Number WCLFU (median, range) | 34.5 (7-109) | 5 (0-56) | 1.11 | 1.05-1.17 | **<0.001** |
| WCLFU as percent of total sample size (median, range) | 5.6% (0.3-16.2%) | 0.8% (0=5.8%) | 2.1 | 1.49-2.95 | **<0.001** |
| Fragility Index as proportion of experimental group size (median, range) | 3.95% (0.08-27.63%) | 11.17% (1.12 – 51.72%) | 0.86 | 0.78-0.95 | **0.002** |
| Sample Size, total (median, range) | 696 (123-4804) | 582 (120-1486) | 1.00 | 0.99-1.00 | 0.18 |
| Year of Approval (n, %)  2010/12 (n=16)  2013/15 (n=20)  2016/17 (n=16)  2018/19 (n=19) | 7 (19.4%)  10 (27.8%)  12 (33.3%)  7 (19.4%) | 9 (25.7%)  10 (28.6%)  4 (11.4%)  12 (24.3%) | 1.00  1.28  3.86  0.75 | 0.34-4.81  0.86-17.32  0.19-2.92 | 0.95^*^ |
| Number of Events Total (median, range) (n=67) | 334 (56-884) | 290 (81-863) | 1.00 | 0.99-1.00 | 0.39 |
| Drug Class (n, %)  Immunotherapy (n=18)  Chemotherapy (n=5)  Monocolonal Antibodies (n=12)  Androgen Receptor Blockers (n=2)  TKI (n=21)  Targeted (n=11)  Other (n=2) | 10 (27.8%)  2 (5.6%)  9 (25.0%)  1 (2.8%)  9 (25.0%)  4 (11.1%)  1 (2.8%) | 8 (22.9%)  3 (8.6%)  3 (8.6%)  1 (2.7%)  12 (34.3%)  7 (20.0%)  1 (2.9%) | 1.00  0.53  2.4  0.8  0.6  0.45  0.8 | 0.07-2.01  0.48-11.93  0.04-14.89  0.17-2.13  0.10-2.13  0.04-14.89 | 0.54  0.28  0.88  0.43  0.32  0.88 |
| Setting of Approval (n, %)  Metastatic (n=63)  Adjuvant (n=8) | 30 (83.3%)  6 (16.7%) | 33 (94.3%)  2 (5.7%) | 1.00  3.3 | 0.62-17.6 | 0.16 |
| Malignancy Site (n, %)  Breast (n=14)  Lung (n=19)  Melanoma (n=7)  Prostate (n=3)  Other (n=28) | 9 (25.0%)  9 (25.0%)  5 (13.9%)  1 (2.8%)  12 (33.3%) | 5 (14.3%)  10 (28.6%)  2 (5.7%)  2 (5.7%)  16 (45.7%) | 1.00  0.50  1.39  0.28  0.42 | 0.12-2.06  0.19-9.97  0.02-3.88  0.11-1.56 | 0.34  0.74  0.34  0.19 |
| Endpoint (n, %)  Other (n=41)  OS (n=30) | 18 (50.0%)  18 (50.0%) | 23 (65.7%)  12 (34.3%) | 1.00  1.91 | 0.73-4.98 | 0.18 |
| Difference in time to event outcome between intervention and control in months (median, range) (n=55) | 3.3 (1.2-15.3) | 4.2 (1.4-14.6) | 0.99 | 0.84-1.16 | 0.92 |
| Approval Pathway (n, %)  Regular approval (n=67)  Accelerated Approval (n=4) | 32 (88.9%)  4 (11.1%) | 35 (100%)  0 | 1.00  - | - | - |
| Breakthrough Therapy Designation (n, %)  No (n=36)  Yes (n= 21)  N/A (n=14) | 19 (52.8%)  10 (27.8%)  7 (19.4%) | 17 (48.6%)  11 (31.4%)  7 (20.0%) | 1.00  0.81  0.89 | 0.28-2.39  0.26-3.08 | 0.71  0.86 |
| Fast Track (n, %)  No (n=58)  Yes (n=8)  N/A (n=5) | 30 (83.3%)  4 (11.1%)  2 (5.6%) | 28 (80.0%)  4 (1.4%)  3 (8.6%) | 1.00  0.93  0.62 | 0.21-4.09  0.10-4.00 | 0.93  0.62 |
| Priority Review (n, %)  No (n169)  Yes (n=55) | 8 (22.2%)  29 (77.8%) | 8 (22.9%)  27 (77.1%) | 1.00  1.04 | 0.34-3.16 | 0.95 |
| Control Group (n, %)  Active Control (n=59)  Placebo or BSC alone (n=12) | 29 (80.6%)  7 (19.4%) | 30 (85.7%)  5 (14.3%) | 1.00  1.45 | 0.41-5.08 | 0.56 |

Abbreviation: BSC, best supportive care; FU, follow up; N/A, not available; OR, odds ratio; CI, confidence interval; FI, fragility index; OS; overall survival; TKI, Tyrosine kinase inhibitors.

^*^ p test for trend

^**^ While the HR are reported based on the trial results, the OR and P values are reported for the log transformed HR.

**Supplemental Table 6: Association Between Studies where Early Drug Discontinuation Exceeds the Fragility Index and Trial Characteristics (n=117)**

|  | Early Drug Discontinuation > than FI  (n=76)  (code 1) | Early Drug Discontinuation < than FI (n=41)  (code 0) | OR | 95% CI | P |
| --- | --- | --- | --- | --- | --- |
| Fragility Index (median, range) | 12.5 (1-254) | 48 (7-322) | 0.96 | 0.95-0.98 | **<0.001** |
| Number with Early Drug Discontinuation (median, range) | 51.5 (6-600) | 22 (4-132) | 1.04 | 1.02-1.07 | **<0.001** |
| Reported HR (median, range) (n=114)^**^ | 0.67 (0.37-0.86) | 0.47 (0.21-0.74) | 207.15 | 24.56-1747.45 | **<0.001** |
| Fragility Index as proportion of experimental group size (median, range) | 5.3% (0.08-27.63%) | 16.2% (3.7-51.7%) | 0.78 | 0.71-0.86 | **<0.001** |
| Sample Size (median, range) | 666.5 (123-4804) | 493 (117-1401) | 1.00 | 0.99-1.00 | **0.07** |
| Year of Approval (n, %)  2010/12 (n=23)  2013/15 (n=31)  2016/17 (n=25)  2018/19 (n=38) | 15 (19.7%)  21 (27.6%)  17 (22.4%)  23 (30.3%) | 8 (19.5%)  10 (24.4%)  8 (19.5%)  15 (36.6%) | 1.00  1.12  1.13  0.82 | 0.35-3.5  0.34-3.77  0.28-2.40 | 0.65^*^ |
| Number of Events (median, range) (n=107) | 295 (56-884) | 290 (81-1077) | 1.00 | 0.99-1.00 | 0.48 |
| Drug Class (n, %)  Immunotherapy (n=26)  Chemotherapy (n=9)  Monocolonal Antibodies (n=17)  Androgen Receptor Blockers (n=6)  TKI (n=31)  Targeted (n=25)  Other (n=3) | 20 (26.3%)  5 (6.6%)  14 (18.4%)  3 (3.9%)  18 (23.7%)  14 (18.4%)  2 (2.6%) | 6 (14.6%)  4 (9.8%)  3 (7.3%)  3 (7.3%)  13 (31.7%)  11 (26.8%)  1 (2.4%) | 1.00  0.37  1.40  0.30  0.41  0.38  0.60 | 0.07-1.86  0.30-6.56  0.05-1.89  0.13-1.32  0.11-1.28  0.05-7.82 | 0.23  0.67  0.20  0.14  0.12  0.70 |
| Setting of Approval (n, %)  Metastatic (n=106)  Adjuvant (n=11) | 68 (89.5%)  8 (10.5%) | 38 (92.7%)  3 (7.3%) | 1.00  1.49 | 0.37-5.95 | 0.57 |
| Malignancy Site (n, %)  Breast (n=21)  Lung (n=25)  Melanoma (n=13)  Prostate (n=8)  Other (n=50) | 15 (19.7%)  14 (18.4%)  10 (13.2%)  5 (6.6%)  32 (42.1%) | 6 (14.6%)  11 (26.8%)  3 (7.3%)  3 (7.3%)  18 (43.9%) | 1.00  0.51  1.33  0.67  0.71 | 0.14-1.74  0.27-6.61  0.11-3.71  0.23-2.16 | 0.28  0.72  0.64  0.55 |
| Endpoint (n, %)  Other (n=71)  OS (n=46) | 35 (46.1%)  41 (53.9%) | 36 (87.8%)  5 (12.2%) | 1.00  8.43 | 2.98-23.82 | **<0.001** |
| Difference in time to event outcome between intervention and control in months (median, range) (n=94) | 3.3 (1.2-22) | 5.2 (1.3-24.3) | 0.91 | 0.82-0.99 | **0.046** |
| Approval Pathway (n, %)  Regular approval (n=111)  Accelerated Approval (n=6) | 72 (94.7%)  4 (5.3%) | 39 (95.1%)  2 (4.9%) | 1.00  1.08 | 0.19-6.18 | 0.93 |
| Breakthrough Therapy Designation (n, %)  No (n=71)  Yes (n= 30)  N/A (n=16) | 44 (57.9%)  20 (26.3%)  12 (15.8%) | 27 (65.8%)  10 (24.4%)  4 (9.8%) | 1.00  1.22  1.84 | 0.50-3.01  0.54-6.29 | 0.65  0.33 |
| Fast Track (n, %)  No (n=86)  Yes (n=24)  N/A (n=7) | 57 (75.0%)  14 (18.4%)  5 (6.6%) | 29 (70.7%)  10 (24.4%)  2 (4.9%) | 1.00  0.71  1.27 | 0.28-1.80  0.23-6.96 | 0.47  0.78 |
| Priority Review (n, %)  No (n=30)  Yes (n=87) | 19 (25.0%)  57 (75.0%) | 11 (26.8%)  30 (73.2%) | 1.00  1.10 | 0.46-2.61 | 0.83 |
| Control Group (n, %)  Active Control (n=83)  Placebo or BSC alone (n=34) | 57 (75.0%)  19 (25.0%) | 26 (63.4%)  15 (36.6%) | 1.00  0.58 | 0.25-1.31 | 0.19 |

Abbreviation: BSC, best supportive care; FU, follow up; N/A, not available; OR, odds ratio; CI, confidence interval; FI, fragility index; OS; overall survival; TKI, Tyrosine kinase inhibitors.

^*^ p test for trend

^**^ Only 114 included studies as 3 had dichotomous endpoints. While the HR are reported based on the trial results, the OR and P values are reported for the log transformed HR

**Supplemental Figure 1: Association between FI and WCLFU (n=117)**

**
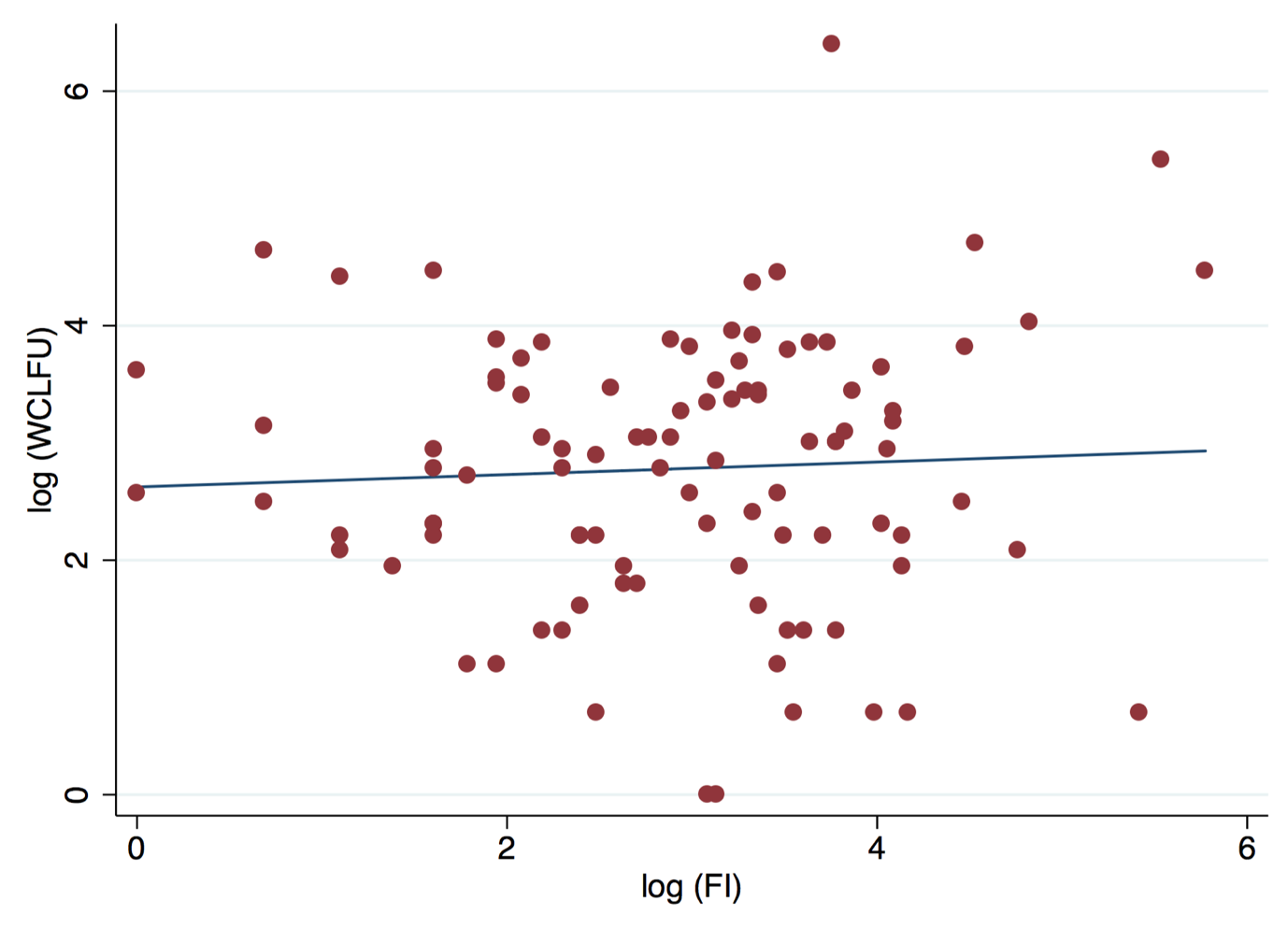
**
